# Detection of Ghost Introgression Requires Exploiting Topological and Branch Length Information

**DOI:** 10.1093/sysbio/syad077

**Published:** 2024-01-15

**Authors:** Xiao-Xu Pang, Da-Yong Zhang

**Affiliations:** Ministry of Education Key Laboratory for Biodiversity Science and Ecological Engineering, College of Life Sciences, Beijing Normal University, Beijing 100875, China; Ministry of Education Key Laboratory for Biodiversity Science and Ecological Engineering, College of Life Sciences, Beijing Normal University, Beijing 100875, China

**Keywords:** BPP, full-likelihood, ghost introgression, heuristic methods, simulation

## Abstract

In recent years, the study of hybridization and introgression has made significant progress, with ghost introgression—the transfer of genetic material from extinct or unsampled lineages to extant species—emerging as a key area for research. Accurately identifying ghost introgression, however, presents a challenge. To address this issue, we focused on simple cases involving 3 species with a known phylogenetic tree. Using mathematical analyses and simulations, we evaluated the performance of popular phylogenetic methods, including HyDe and PhyloNet/MPL, and the full-likelihood method, Bayesian Phylogenetics and Phylogeography (BPP), in detecting ghost introgression. Our findings suggest that heuristic approaches relying on site-pattern counts or gene-tree topologies struggle to differentiate ghost introgression from introgression between sampled non-sister species, frequently leading to incorrect identification of donor and recipient species. The full-likelihood method BPP uses multilocus sequence alignments directly—hence taking into account both gene-tree topologies and branch lengths, by contrast, is capable of detecting ghost introgression in phylogenomic datasets. We analyzed a real-world phylogenomic dataset of 14 species of *Jaltomata* (Solanaceae) to showcase the potential of full-likelihood methods for accurate inference of introgression.

The growing availability of genomic data has led to an acceleration in research on hybridization and subsequent genetic exchange between species (i.e., introgression), which are widely recognized as significant factors in adaptation, speciation, and evolutionary innovation (Mallet et al. 2016; Taylor and Larson 2019; Edelman and Mallet 2021). In addition to ancient and contemporary gene flow between extant lineages extensively reported in the literature (Fontaine et al. 2015; Figueiró et al. 2017; Jones et al. 2018; Wu et al. 2018a; Zhang et al. 2019; Wang et al. 2020; Esquerré et al. 2021; Meleshko et al. 2021; Yang et al. 2021; Suvorov et al. 2022), the phenomenon of “ghost introgression,” which refers to gene flow from extinct or unsampled lineages to the sampled species, is gradually emerging as an important cutting-edge research topic (Ottenburghs 2020; Hibbins and Hahn 2022a; Tricou et al. 2022a, 2022b; Pang and Zhang 2023; Tiley et al. 2023). As the overwhelming majority of lineages have either gone extinct or have been unsampled due to technical limitations or irrelevance to specific research questions, evolutionary studies are inherently constrained to small subsets of species or populations. Ghost introgression, therefore, is a crucial factor to be reckoned with in such studies. Genomic data analyses have provided evidence of ghost introgression in plants (e.g., Ding et al. 2022; Li et al. 2022; Tiley et al. 2023) and animals (e.g., Ai et al. 2015; Kuhlwilm et al. 2019; Rocha et al. 2022), including humans (Green et al. 2010; Sankararaman et al. 2014).

Various phylogenetic methods have been developed over the past few years for detecting and characterizing introgression, ranging in complexity from heuristic methods based on summary statistics to full-likelihood techniques that utilize all the information present in multilocus sequence data (for the latest reviews, see Jiao et al. 2021; Hibbins and Hahn 2022a). Most of these methods use data from one sample per species, even when multiple samples are available (Hibbins and Hahn 2022a). These techniques were designed primarily to detect introgression between sampled taxa, without considering the possibility of ghost introgression (Tricou et al. 2022a, 2022b). Evaluating these methods’ ability to detect ghost introgression will help researchers avoid misinterpretation of results and expand these tools for detecting different types of gene exchange.

The most popular heuristic methods, such as the D-statistic (Green et al. 2010) and HyDe (Blischak et al. 2018), rely on summary statistics obtained from the site-pattern counts for a species quartet to test the presence of gene flow between non-sister species. However, Tricou et al. (2022b) have shown that outgroup ghost introgression, defined as introgression from an unsampled lineage that diverged before the early diverging species under investigation (as illustrated in Fig. 1a), can significantly affect D-statistic values. This can lead to incorrect identification of both donor and recipient species involved in a ghost introgression event. In an attempt to differentiate ghost introgression from other gene flow scenarios, Hibbins and Hahn (2022a) proposed using an additional species that is sister to the recipient but not implicated in any introgression as a reference. However, finding such a suitable reference species can be challenging, making this solution difficult to apply in practice. The HyDe test is based on a model of hybrid speciation and has been used as a well-justified approach for assessing introgression in general (Kong and Kubatko 2021). However, findings by Ji et al. (2023) suggest that the accuracy of HyDe may be compromised when inferring the donor and recipient of introgression events in outflow scenarios, where gene flow occurs from a sister species to the early diverging species (as illustrated in Fig. 1c). The behavior of HyDe under ghost introgression scenarios (Fig. 1a) has yet to be explored.

**Figure 1.**
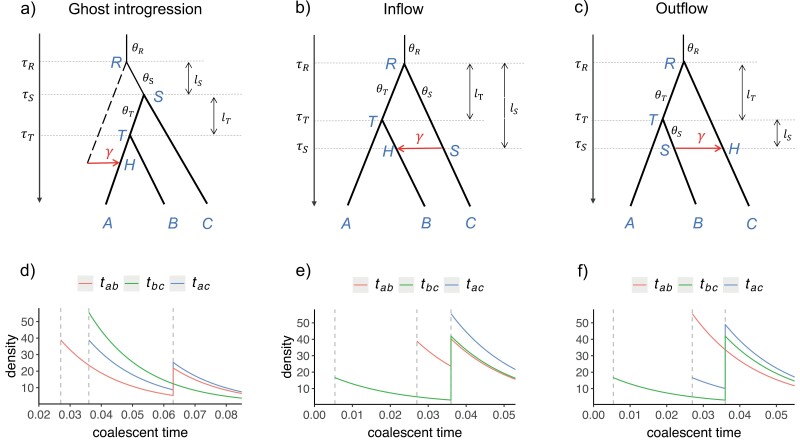
Three different introgression scenarios within a species tree *AB|C* and the corresponding distribution of coalescent times $\left(t_{ab},t_{bc},t_{ac}\right)$ for sequence pairs. a) Introgression from an outgroup ghost to sister species *A* (ghost introgression). b and c) Introgression between non-sister species in different directions *C→B* (inflow) and *B→C* (outflow). The backbone tree is represented by thick branches, the introgression event is labeled by a arrow, and the hybrid species *H* consists of a proportion γ of introgressed genes. $l_{T}$ and $l_{S}$ represent the branch length of the ancestral species *T* and *S*, respectively. Note that the branch lengths of the networks are not to scale. d–f) The distribution of coalescent times $\left(t_{ab},t_{bc},t_{ac}\right)$ for sequence pairs in the introgression scenarios is shown above in Figure 1a–c. In the ghost introgression scenario, the parameters are set as: $\left\{\theta_{S}=\theta_{T}=\theta_{R}=\theta =0.036,\tau_{T}=0.75\theta ,\tau_{S}=\theta ,\tau_{R}=1.75\theta ,\gamma =0.3\right\}$. The vertical dashed lines from left to right in Figure 1d represent the values of $\tau_{T}$, $\tau_{S}$, and $\tau_{R}$, respectively. In the inflow and outflow cases, the parameters are set as: $\left\{\theta_{S}=\theta_{T}=\theta_{R}=\theta =0.036,\;\tau_{S}=0.15\theta ,\tau_{T}=0.75\theta ,\tau_{R}=\theta ,\;\gamma =0.3\right\}$. The vertical dashed lines from left to right in Figure 1e–f represent the values of $\tau_{S}$, $\tau_{T}$, and $\tau_{R}$, respectively.

Another class of approaches is designed to detect introgression across an entire phylogeny by estimating a phylogenetic network on the full set of taxa under investigation. Heuristic methods first summarize sequence data by estimating gene trees and then take gene trees as inputs for constructing phylogenetic networks (Jiao et al. 2021; Cao et al. 2022). Most of them solely utilize gene-tree topologies (or subtree topologies for subsets of species trios or quartets) under a (pseudo-)likelihood or distance-based framework. Examples of such methods include InferNetwork_MPL and InferNetwork_ML in the PhyloNet program (Yu et al. 2014; Yu and Nakhleh 2015), SNaQ in the PhyloNetworks package (Solís-Lemus and Ané 2016), and NANUQ in the MSCquartets package (Allman et al. 2019). However, these heuristic methods have a significant drawback: networks may not always be identifiable. That is to say, the information derived solely from gene-tree topologies may be insufficient to differentiate between networks representing different biological hypotheses about speciation and introgression (Pardi and Scornavacca 2015; Zhu and Degnan 2017; Yang and Flouri 2022). One important thing to note here is that the ML method InferNetwork_ML can take advantage of gene-tree branch lengths (coalescent times); however, it is generally discouraged to do so due to the method’s susceptibility to random sampling errors in branch-length estimates (Cao et al. 2019). Full-likelihood phylogenetic network methods refer to those that operate on multilocus sequence data directly and use the joint distribution of gene-tree topologies and coalescent times (Jiao et al. 2021). These methods utilize all the information in gene-tree topologies and branch lengths and account for gene-tree uncertainties appropriately by averaging over all their probabilities. As a result, they significantly improve the statistical power in identifying networks, but at the expense of a much-increased computational burden (Hey et al. 2018; Wen and Nakhleh 2018; Zhang, Ogilvie, et al. 2018; Flouri et al. 2020). The Bayesian Phylogenetics and Phylogeography (BPP) program (Flouri et al. 2020) is notable for allowing users to compare 2 putative networks using Bayes factors, avoiding cross-model search in large network space. Thus, it is much more computationally feasible.

Here, we examine the effectiveness of various phylogenetic techniques in detecting ghost introgression through a study of 3 introgression scenarios that can lead to the same significant D-statistic, as first made explicit by Tricou et al. (2022b). These scenarios include outgroup ghost introgression (Fig. 1a) and ingroup introgression between non-sister species, with either C→B (inflow, Fig. 1b) or B→C (outflow, Fig. 1c) directions within the species tree *AB|C*. Our analysis is limited to cases where only one sequence is sampled from each species, as the addition of more samples may provide little new information regarding introgression (Hibbins and Hahn 2022a; but see Huang et al. 2022 for a dissenting view). We begin by mathematically deriving probabilities of gene-tree topologies, frequencies of biallelic site patterns, and distributions of coalescent times for pairs of sequences from different species. This allows us to analyze how well these pieces of information can distinguish outgroup ghost introgression from other introgression scenarios. We then conduct simulations to examine the behaviors of site pattern-based HyDe, gene tree-based InferNetwork_MPL in PhyloNet (or simply PhyloNet/MPL), and the full-likelihood method BPP under the aforementioned introgression scenarios. As a real-world example, we analyze a phylotranscriptomic dataset of 14 *Jaltomata* (Solanaceae) species (Wu et al. 2018; Tiley et al. 2023) to demonstrate the potential pitfalls of using heuristic methods as well as the feasibility of using full-likelihood methods to detect ghost introgression in mid-sized groups of lineages of interest. By integrating theoretical analysis, simulation evaluation, and practical examples, our study provides valuable insights for biologists investigating ghost introgression, highlighting which methods are most suitable for this very purpose.

## Theory

We consider 3 gene flow scenarios, likely associated with a significant D-statistic (Tricou et al. 2022b), within the species tree *AB|C* (along with a distant outgroup *O*) under the model of multispecies coalescent with introgression (MSci; Yu et al. 2012; Flouri et al. 2020). These scenarios include outgroup ghost introgression involving gene flow from an outgroup ghost lineage to species *A* (Fig. 1a, referred to as ghost introgression hereafter), as well as ingroup introgression between non-sister species *B* and *C* in different directions (inflow and outflow, depicted in Fig. 1b and c). Both species divergence times $\left(\tau_{R},\tau_{T},\tau_{S}\right)$ and population sizes $\left(\theta_{R},\theta_{T},\theta_{S}\right)$ in the models are quantified using the expected number of mutations per site as the measurement unit. Specifically, the divergence time is defined as τ=Tμ, where *T* is the divergence time in generations and μ is the mutation rate per site per generation. The population size refers to θ=4Nμ, where *N* is the number of diploid individuals in the species. The data analyzed consists of multiple loci, with 3 sequences from each species at each locus (a,b,c). The possible gene-tree topologies at each locus are $G_{1}=ab|c$, $G_{2}=a|bc$, and $G_{3}=ac|b$.

Note that the 3 scenarios of ghost introgression, inflow, and outflow share the same speciation history of *AB|C* and the introgressed history of *A|BC*, despite having distinct recipients of gene flow—*A*, *B*, and *C*, respectively. As a result, the 3 introgression scenarios can produce similar features in gene genealogies and sequence data. Our main focus is to examine to what extent the 3 introgression scenarios can be distinguished with different types of information. Specifically, we derive analytical results in network identification and the estimation of introgression probability γ for 3 methods: 1) PhyloNet/MPL, which is based on probabilities of gene-tree topologies, 2) HyDe, which relies on site-pattern frequencies, and 3) full-likelihood methods, which use the joint distribution of gene-tree topologies and coalescent times.

### Identifiability Based on Probabilities of Gene-tree Topologies (Analysis of PhyloNet/MPL)

In the 3 MSci models shown in Fig. 1, we define $C_{T}=2l_{T}/\theta_{T}$ and $C_{S}=2l_{S}/\theta_{S}$ as the lengths, in coalescent units, of the internal branch *T* in the species history and the internal branch *S* in the introgression history, respectively. Following the speciation history *AB|C* with a probability of 1−γ, if sequences *a* and *b* coalesce in species *T*, the gene tree would be $G_{1}=ab|c$; following the introgressed history *A|BC* with a probability of γ, if sequences *b* and *c* coalesce in species *S*, the gene tree would be $G_{2}=a|bc$. If neither of these events occurs, indicating the occurrence of incomplete lineage sorting (ILS) within either the speciation or introgressed history, then 3 gene trees will occur with equal probability. The probabilities of the 3 gene-tree topologies can be represented using the following equations, which have been derived in Jiao et al. (2020) and Pang and Zhang (2023):


$$\begin{array}{llllll} P(G_{3})=\; & \frac{1}{3}(1-\gamma )e^{-C_{T}}+\frac{1}{3}\gamma e^{-C_{S}}\;P(G_{1})=\; & (1-\gamma )\left(1-\frac{2}{3}e^{-C_{T}}\right)+\frac{1}{3}\gamma e^{-C_{S}}\;=\; & (1-\gamma )(1-e^{-C_{T}})+P(G_{3})\;P(G_{2})=\; & \frac{1}{3}(1-\gamma )e^{-C_{T}}+\gamma \left(1-\frac{2}{3}e^{-C_{S}}\right)\;=\; & \gamma (1-e^{-C_{S}})+P(G_{3}). \end{array}$$ (1)

The sum of probabilities of 3 gene-tree topologies equals 1, leaving only 2 free quantities: $P(G_{1})-P(G_{3})=(1-\gamma )(1-e^{-C_{T}})$ and $P(G_{2})-P(G_{3})=\gamma (1-e^{-C_{S}})$. It is worth noting that these expressions for probabilities of gene-tree topologies can be applied to all 3 introgression scenarios. This means that gene-tree topologies can have identical probabilities across the 3 introgression scenarios, provided that the values of $\left(1-\gamma )(1-e^{-C_{T}}\right)$ and $\gamma (1-e^{-C_{S}})$ remain invariant across scenarios. As a consequence, the PhyloNet/MPL method that uses information only from gene-tree topologies cannot differentiate between the scenarios of ghost introgression, inflow, and outflow. Moreover, the limitation persists even when multiple sequences per species are available. This is because PhyloNet/MPL only considers sequence permutations within 3 species, selecting one sequence from each species. Consequently, this approach for accommodating multiple samples per species results in only 3 gene-tree topologies without providing more information for improving network identification.

Note that a similar limitation applies to another popular heuristic method, known as SNaQ in the PhyloNetworks program (Solís-Lemus and Ané 2016; Solís-Lemus et al. 2017), which employs unrooted quartets for network inference. This is because Equation (1) remains valid when assigning $G_{1}=ab|co$, $G_{2}=bc|ao$_,_ and $G_{3}=ac|bo$. In other words, SNaQ encounters the same issue as PhyloNet/MPL; it also cannot effectively distinguish between the scenarios of ghost introgression, inflow, and outflow.

Furthermore, we examine the estimation of introgression probability γ. With only 2 independent equations, there are infinite solutions for the 3 unknown parameters $\left\{\gamma ,C_{T},C_{S}\right\}$. Specifically, given the observed gene-tree topology probabilities $P(G_{1})$, $P(G_{2})$_,_ and $P(G_{3})$, any value of γ within the range of $P(G_{2})-P(G_{3})<\gamma <1-(P(G_{1})-P(G_{3}))$ can find corresponding solutions for $C_{T}$ and $C_{S}$ to satisfy the 2 equations. By substituting the expressions for $P(G_{i})\;\left\{i=1,\;2,\;3\right\}$ from Equation (1), it can be shown that the PhyloNet/MPL estimates of γ, denoted as $\gamma_{Phy}$, will fall within this range:


$$\begin{array}{l} \begin{array}{l} \gamma -\gamma e^{-C_{S}}<\gamma_{Phy}<\gamma +(1-\gamma )e^{-C_{T}}\; \end{array}. \end{array}$$ (2)

**Figure 2.**
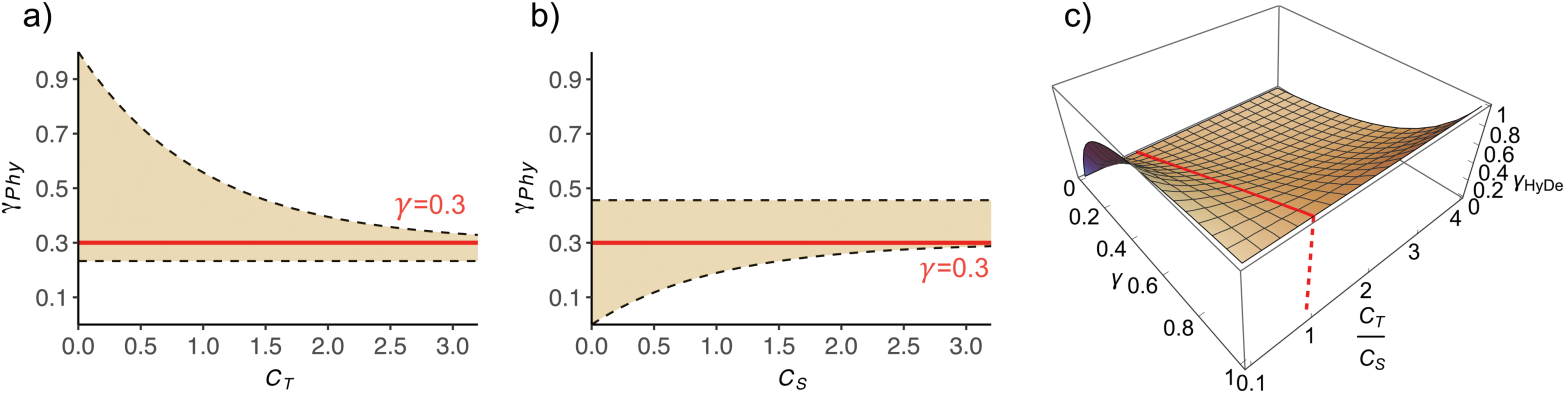
Estimation of introgression proportion γ in PhyloNet/MPL and HyDe. In reference to the introgression scenarios illustrated in Figure 1, $C_{T}=2l_{T}/\theta_{T}$ and $C_{S}=2l_{S}/\theta_{S}$ denote the branch lengths in coalescent units of ancestral species *S* and *T*, respectively. a and b) In PhyloNet/MPL, when γ=0.3 labeled by the thick solid line, the estimate $\gamma_{Phy}$ would disperse over the range demarcated by the light-shaded area, with the upper and lower bounds labeled by the dashed lines. a) The range for $\gamma_{Phy}$ varies with the value of $C_{T}$ after fixing $C_{S}=1.5$. b) The range for $\gamma_{Phy}$ varies with the value of $C_{S}$ after fixing $C_{T}=1.5$. Obviously, the upper and lower limits for the estimate $\gamma_{Phy}$ depend, respectively, on the value of $C_{T}$ and $C_{S}$. c) In HyDe, under the assumption of constant population size, the estimate $\gamma_{HyDe}$ is related to the ratio $C_{T}/C_{S}$. When $C_{T}=C_{s}$, HyDe can accurately estimate the value of γ, as demonstrated by the thick line. Otherwise, γ may be underestimated or overestimated, depending on the relative values of $C_{T}$ and $C_{S}$.

Obviously, the upper and lower bounds for $\gamma_{Phy}$ depend, respectively, on the value of $C_{T}$, reflecting the level of ILS within the speciation history, and the value of $C_{S},$ reflecting the level of ILS within the introgressed history (Fig. 2a, b). The larger values of $C_{T}$ and $C_{S}$, and hence the weaker ILS in both histories, the more precise estimation of *γ* will be achieved. However, when both $C_{T}$ and $C_{S}$ approach zero, the estimated *γ* will become highly variable within the range of 0 to 1 (Fig. 2a, b).

### Identifiability Based on Site Patterns (Analysis of HyDe)

Assuming an infinite-site mutation model (i.e., no multiple hits), 3 biallelic site patterns, BBAA, ABBA, and BABA (with A representing the ancestral state from the outgroup listed last and B the derived state), are generated through mutations on the internal branches of gene trees $G_{1}$, $G_{2}$, and $G_{3}$, respectively. Thus, the expected number of the 3 parsimony-informative sites can be indicated by the sum of the lengths of internal branches in the relevant gene trees (Mendes and Hahn 2018; Hibbins and Hahn 2022b). Specifically, we weigh the internal branch lengths of the relevant gene trees by their probabilities and sum them across coalescent histories (further details are provided in Supplementary Note 1 available on Dryad at https://doi.org/10.5061/dryad.zs7h44jfz). Here, we derive the frequency of each site pattern per nucleotide site under the assumption of a constant population size with a common θ:


$$\begin{array}{llllll} f(BABA)=\; & \frac{\theta }{6}(1-\gamma )e^{-2l_{T}/\theta }+\frac{\theta }{6}\gamma e^{-2l_{S}/\theta }\;f(BBAA)=\; & (1-\gamma )l_{T}+\frac{\theta }{6}(1-\gamma )e^{-2l_{T}/\theta }+\frac{\theta }{6}\gamma e^{-2l_{S}/\theta }\;=\; & (1-\gamma )l_{T}+f(BABA)\;f(ABBA)=\; & \gamma l_{S}+\frac{\theta }{6}(1-\gamma )e^{-2l_{T}/\theta }+\frac{\theta }{6}\gamma e^{-2l_{S}/\theta }\;=\; & \gamma l_{S}+f(BABA).\; \end{array}$$ (3)

Hibbins and Hahn (2022b) have derived similar equations by considering branch lengths in coalescent units. Notably, these expressions are universally applicable to all 3 introgression scenarios, where the parameters $l_{T}$ and $l_{S}$ denote the lengths of the internal branches within the speciation and the introgressed histories, respectively, in each scenario. In all 3 scenarios, the site patterns are ordered in the same way, that is, f(BABA)<min{f(BBAA),f(ABBA)}.

HyDe executes the statistical test for introgression detection, with the null hypothesis being the MSC model where the 2 least frequent site patterns are equal (Kubatko and Chifman 2019). Thus, the effectiveness of HyDe in detecting introgression is related to the value of min{f(BBAA)−f(BABA),f(ABBA)−f(BABA)}, that is, $min\;\{\;(1-\gamma )l_{T},\;\gamma l_{S}\;\}\;$, with a larger value indicating greater ease of detection. HyDe interprets a significant outcome as resulting from a hybrid speciation event, in which the 2 parent species are expected to be more distantly related than either of them to the hybrid. Thus, the 2 species that share the derived state in the smallest number of site patterns will be identified as putative parents, and the remaining species as a hybrid (Kubatko and Chifman 2019). In the 3 distinct introgression scenarios in Figs 1a–c, the site pattern BABA is always the least numerous; as a result, HyDe will invariably point to an inflow scenario with species *B* being the hybrid. Finally, HyDe estimates γ using the function (Blischak et al. 2018):


$$\begin{array}{lllll} \gamma_{HyDe}= & \;\frac{f\;\left(ABBA\right)-f\;\left(BABA\right)}{f\;\left(ABBA\right)+f\;\left(BBAA\right)-2f\;\left(BABA\right)}\; & \;=\;\frac{\gamma l_{S}}{\gamma l_{S}+\left(1-\gamma \right)l_{T}}\; & \;=\frac{1}{1+\left(1/\gamma -1\right)l_{T}/l_{S}}\; & =\;\frac{1}{1+\left(1/\gamma -1\right)C_{T}/C_{S}}\; \end{array}$$ (4)

The estimation of γ is tied to $l_{T}/l_{S}$ or $C_{T}/C_{S}$. Accurate estimation of γ can only be achieved when $l_{T}=l_{S}$ (or $C_{T}=C_{S}$). Otherwise, the value of γ will either be underestimated or overestimated, depending on whether the ratio of $l_{T}/l_{S}$ (or $C_{T}/C_{S}$) is larger or smaller than 1 (Fig. 2c).

### Network Identifiability of Full-Likelihood Methods

Full-likelihood methods use the joint probability distribution of gene-tree topologies and coalescent times (i.e., gene-tree branch lengths). For ease of analysis, we focus on the distributions of coalescent times between any 2 sequences, namely, $t_{ab}$, $t_{bc}$, and $t_{ac}$ (Fig. 1d–f). In a population of size θ, the coalescent time of 2 sequences follows an exponential distribution with a mean of θ/2. The probability densities of coalescent times for the 3 introgression scenarios are derived in Supplementary Note 2 and plotted for specific parameters in Figure 1d–f.

There are 2 unique characteristics of coalescent times for each scenario. Firstly, the order of the minimum coalescence times for the 3 sequence pairs differs across the three cases. Specifically, in the ghost introgression scenario, the minimum coalescence times of sequence pairs align with the original species tree, where $min(t_{ab})<min(t_{ac})=min(t_{bc})$ (Fig. 1d). In the inflow case, introgression reduces the minimum coalescent time of the sequence pair *bc* from the root time to the introgression time, while the minimum coalescent times for the other two sequence pairs remain unaffected. As a result, the order becomes $min(t_{bc})<min(t_{ab})<min(t_{ac})$ (Fig. 1e). In the outflow case, introgression reduces the minimum coalescent times for sequence pairs *ac* and *bc* from the root time to the speciation time of the sister species and to the more recent introgression time, respectively, resulting in the order of $min(t_{bc})<min(t_{ab})=min(t_{ac})$ (Fig. 1f).

Secondly, the distribution form of coalescence times for sequence pairs varies among the 3 scenarios of ghost introgression, inflow, and outflow, each of which involves a specific recipient: *A*, *B*, and *C*, respectively. The coalescent time of the sequences of the recipient and another species will have a mixture distribution that is discontinuous at certain time points because the sequence of the recipient lineage has 2 coalescence paths. However, introgression does not affect the coalescent process for a pair of sequences from the 2 species not acting as the recipient, and the coalescent time follows a smooth shifted exponential distribution when constant population size is assumed. Thus, using one sequence per species, full-likelihood methods are capable of distinguishing among the 3 scenarios.

## Simulations

### Data Simulation for Evaluation of Introgression Detecting Methods

**Figure 3.**
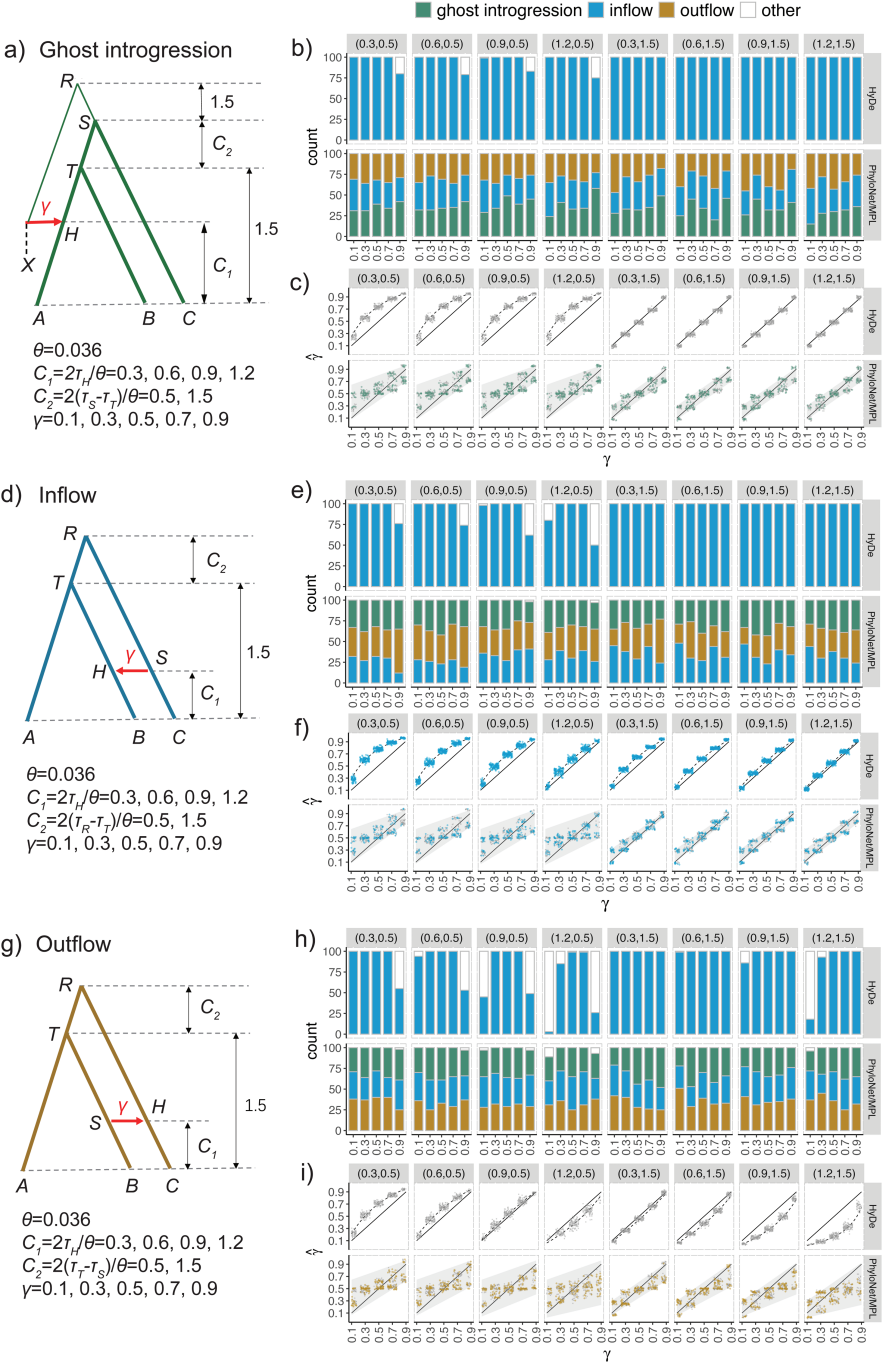
Results of HyDe and PhyloNet/MPL for the scenarios of ghost introgression, inflow, and outflow. a) Ghost introgression scenarios with corresponding parameter settings. b and c) Plots of results for network inference and γ estimation for ghost introgression simulations. The strips located on the top and right of each plot represent branch lengths in coalescent units $\left(C_{1},C_{2}\right)$ and the employed methods, respectively. The *x*-axis indicates the value of γ. b) Estimation of network topologies. The numbers of 3 network models (i.e., ghost introgression, inflow, and outflow) inferred by HyDe and PhyloNet/MPL (using the command InferNetwork_MPL) among 100 replicates are represented by bars. The other networks correspond to statistically insignificant results (B–H-adjusted *P* value < 0.05) in HyDe or networks other than those three in PhyloNet/MPL. c) Estimation of introgression probability γ by HyDe and PhyloNet/MPL. Colored and gray points represent the estimates of γ in the true and other 2 false networks, respectively. These points are horizontally jittered to avoid clutter. The solid line shows the true values, while the dashed line displays the expected estimates by HyDe based on Equation (4) in the main text, and the gray-shaded area represents the expected range of the estimates by PhyloNet/MPL according to Equation (2) in the main text. d–f) Inflow scenarios and corresponding simulation results. g–i) Outflow scenarios and corresponding simulation results.

We simulated 3 scenarios—ghost introgression, inflow, and outflow—with varying levels of ILS within the speciation history $\left(C_{2}\right)$, as well as varying levels (γ) and times $\left(C_{1}\right)$ of introgression, as shown in Fig. 3. For each case, we set the linking of the divergence of the outgroup species *O* to *R* to 5 coalescent units, and we simulated 100 replicates. We employed *ms* simulator (Hudson 2002) to generate 1000 gene trees with one sequence per species. These gene trees were then used to simulate DNA sequences of 1000 base pairs by Seq-Gen (Rambaut and Grass 1997) with the options -s 0.036 (as in Yu et al. 2014; Kong and Kubatko 2021) and -m HKY. The concatenated sequences were used for HyDe. Notably, we found that HyDe has a high false positive rate due to the non-independence of sites within a locus sharing the same underlying genealogy (Supplementary Fig. S1). To address this issue, we used the block-jackknife approach to estimate sample variance and then calculate the significance value. We accounted for multiple testing by using the Benjamini–Hochberg False Discovery Rate correction, that is, results were considered significant if the B–H-adjusted *P*-values were less than 0.05 (Supplementary Fig. S1). We rerooted the simulated gene trees using the outgroup *O*, which was then removed. The rooted gene trees were used as inputs for the maximum pseudo-likelihood method InferNetwork_MPL in PhyloNet (v3.8.2) (PhyloNet/MPL). Furthermore, we conducted additional simulations with 4 sequences per species to explore the impact of multiple sequences per taxon on PhyloNet/MPL.

We used the full-likelihood implementation of the MSci model in BPP (v4.6.2) (Flouri et al. 2020) to analyze the sequence data (excluding the outgroup *O*). There are other full-likelihood methods available in literature, including MCMC_SEQ in PhyloNet (Wen and Nakhleh 2018) and SpeciesNetwork in *BEAST (Zhang, Ogilvie, et al. 2018), but they are too computationally demanding to use in the present simulation study. In BPP, we compared ghost introgression, inflow, and outflow scenarios by calculating the marginal likelihoods. Thermodynamic integration combined with Gaussian quadrature was used to calculate the marginal likelihood under each scenario, with 16 quadrature points (Lartillot and Philippe 2006; Rannala and Yang 2017). For each of the 16 MCMC runs, we used 50,000 MCMC iterations as burnin and then took 10^6^ samples, sampling every 2 iterations. Then, we used an A00 analysis of Yang (2015) to estimate the parameters under the true MSci model with the same MCMC iteration settings. Each run took ~6 h using a single thread. Additionally, we pursued an alternative heuristic approach to address gene-tree branch lengths as a comparison. We ran the ML algorithm InferNetwork_ML (Yu et al. 2014) in PhyloNet v3.8.2 (or simply PhyloNet/ML) with the “-bl” option. We prepared two sets of data. The first set consisted of simulated gene trees with accurate topology and branch lengths (in coalescent units). For the second set, we constructed gene trees using IQ-TREE v2 (Minh et al. 2020) and then rerooted them by the outgroup *O*. A scale parameter of 2/*θ* was used to convert branch lengths from the expected number of per-site mutations to the number of coalescent units.

We further evaluated the performance of PhyloNet/MPL in detecting ancient ghost introgression events. We considered ancient introgression scenarios where lineages diverged subsequent to an introgression event (Fig. 5). For each condition, we simulated 20 replicates and generated 1000 gene trees for each replicate with one sequence per species. The gene trees were rerooted by the outgroup *O*. These rooted gene trees without the outgroup were used as inputs for PhyloNet/MPL.

### Results for the Heuristic Methods: HyDe and PhyloNet/MPL

The results of HyDe and PhyloNet/MPL under different combinations of $C_{1}$, $C_{2}$, and γ in ghost introgression scenarios are summarized in Fig. 3a–c. In terms of testing for the presence or absence of introgression (regardless of its type), the sensitivity of HyDe is not influenced by the time of introgression $\left(C_{1}\right)$, but rather by the degree of ILS in the speciation history $\left(C_{2}\right)$ and the level of introgression (γ), as shown in Fig. 3b. A higher ILS in the speciation history (smaller $C_{2}$) diminishes the power of HyDe for introgression detection. Furthermore, in line with theoretical expectations, HyDe incorrectly attributes ghost introgression to inflow. It mistakenly identifies species *C* and *B* as donor and recipients of gene flow, even though they have not been involved in any genetic exchange. The performance of PhyloNet/MPL is also poor, as it has a tendency to randomly infer 3 scenarios of ghost introgression, inflow, and outflow, regardless of the parameter combinations (Fig. 3b). This result is in line with our theoretical analysis, which suggests that the probabilities of gene-tree topologies are not capable of distinguishing between the 3 introgression scenarios. Regarding the introgression probability γ, the estimates in HyDe conform perfectly to the theoretical prediction derived from Equation (4), even though the assumption of an infinite site model is not strictly satisfied in our simulation data (Fig. 3c). The γ estimates obtained from PhyloNet/MPL fall within the range predicted by Equation (2). More specifically, the accuracy of γ estimation in HyDe and PhyloNet is linked to the level of ILS within the speciation history $\left(C_{2}\right)$, as well as the amount of ILS within the introgression history that depends on the distance of the outgroup ghost (i.e., the length of *RS* fixed at 1.5 in Fig. 3a). When ILS is strong in the speciation history with $C_{2}=0.5$, HyDe overestimates γ because $C_{2}<RS$. The estimates from PhyloNet/MPL exhibit multimodality, forming two clusters with a large difference between them. In the cases with $C_{2}=1.5$, HyDe accurately estimates γ, as the levels of ILS within the 2 histories are equal, despite an incorrect inference of the introgression scenario. In PhyloNet/MPL, the difference between the 2 clusters of estimates decreases, and the estimates become closer to the true value. Notably, the dispersion of γ estimation in PhyloNet/MPL is not caused by random errors resulting from insufficient data, but rather by systematic bias arising from parameter unidentifiability.

The results of the inflow scenario are summarized in Fig. 3d–f. HyDe performs well in this case because it accurately identifies the hybrid lineage *B* in almost all replicates except when ILS is prevalent at $\;\;\;C_{2}=0.5$ and γ=0.1, 0.9, in agreement with the results of Kong and Kubatko (2021). By contrast, PhyloNet/MPL performs poorly and cannot distinguish the three scenarios (Fig. 3e). HyDe overestimates the introgression probability γ (Fig. 3f), as the level of ILS within the speciation history is always higher than that within the introgressed history (*RT* < *RS*) under a constant population size. The extent of overestimation is greater when ILS is stronger in the speciation history and the introgression event happened more recently, for example, $\left(C_{1},C_{2})=(0.3,\;0.5\right)$. The precision of γ estimates in PhyloNet/MPL is mainly influenced by the extent of ILS within the speciation history $\left(C_{2}\right)$. When ILS is prevalent such as when $C_{2}=\;0.5$, the estimates are dispersed over a broad range, suffering from high variance. In contrast, the estimates of γ become more precise and stable when $C_{2}=1.5$.

Fig. 3g–i presents the results of the outflow cases. HyDe shows lower support for the existence of introgression (Fig. 3h) in outflow cases than in inflow cases (Fig. 3e). The percentage of replicates supporting introgression is less than 50% in certain cases with high ILS within the speciation history $\left(C_{2}=0.5\right)$ and extreme values of introgression probability (γ=0.1, 0.9). More significantly, HyDe reverses the direction of introgression and misidentifies species *B* as hybrid, as also found in the simulation of Ji et al. (2023). γ is overestimated by HyDe in some cases, such as when $\left(C_{1},C_{2})=(0.3,\;0.5\right)$, and underestimated in other cases, such as when $\left(C_{1},C_{2})=(1.2,\;1.5\right)$, depending on the relative levels of ILS within the speciation $\left(C_{2}\right)$ and introgressed histories (the length of the branch *TS*) (Fig. 3i). When the levels of ILS are close, such as at $\left(C_{1},C_{2})=(0.9,\;0.5\right)$, even though the direction of gene flow between non-sister species is incorrectly inferred, the extent of introgression γ is estimated with high accuracy. PhyloNet/MPL easily confuses outflow with ghost introgression or inflow (Fig. 3h) and exhibits a great degree of variability for estimates of γ at a high level of ILS (Fig. 3i).

We also investigated the performance of PhyloNet/MPL when multiple sequences were used per species, with results summarized in Supplementary Figures S2–S4. The performance of PhyloNet/MPL in such cases was similar to the single-sequence cases. In other words, even when multiple sequences per species are available, PhyloNet/MPL is still unable to differentiate between the 3 introgression scenarios. Thus, we conclude that PhyloNet/MPL generally cannot identify ghost introgression.

### Results for the Full-likelihood Method: BPP

**Figure 4.**
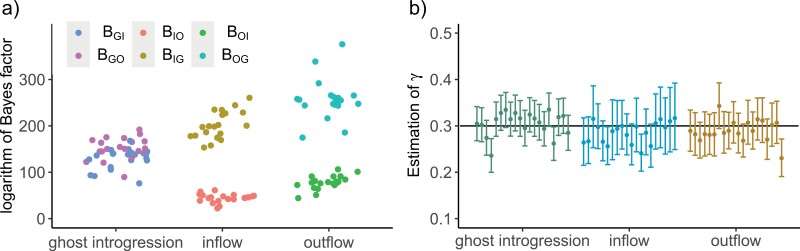
Results of the full-likelihood method BPP. a) The 3 introgression scenarios on *x*-axis are simulated based on the specific parameter combination $\left(C_{1},C_{2},\gamma )=(0.3,\;0.5,\;0.3\right)$. For each simulated dataset, we computed the Bayes factor to compare the 3 models of ghost introgression, inflow, and outflow. The *y*-axis presents the logarithm of Bayes factors, representing the difference in logarithm of marginal likelihoods between the true model and two alternative false models. *B*_*ij*_ refers to the Bayes factor for model *i* against model *j* (G: ghost introgression, I: inflow, O: outflow). In all introgression scenarios, the Bayes factors strongly support the true model across 20 replicate datasets. b) Average posterior means and 95% highest-probability-density credibility intervals for the parameter γ. The black line indicates the true value.

Due to considerable computational requirements, we limited our analysis to the parameter combination $\left(C_{1},C_{2},\gamma )=(0.3,\;0.5,\;0.3\right)$ for 3 scenarios of ghost introgression, inflow, and outflow. We compared 3 network models using Bayes factors through BPP for each simulation dataset, and the results are presented in Fig. 4. For each scenario, it was observed that the true introgression model exhibited substantial superiority over other models, with the logarithm of Bayes factors exceeding 22 across all replicates (Fig. 4a). Moreover, in comparison with heuristic approaches, full-likelihood methods can identify the parameters of species divergence times $\tau_{S}$, ancestral population sizes $\theta_{S}$, and introgression probability γ (Fig. 4b and Supplementary Fig. 5). In all 3 scenarios, the introgression probability γ and species divergence times $\left(\tau_{S},\tau_{T},\tau_{R}\right)$ are well-estimated with narrow confidence intervals. However, it should be noted that the introgression time $\tau_{H}$ cannot be inferred here, but it would become identifiable when using multiple sequences per species. The precision of the estimated population size $\theta_{S}$ varies across different ancestral populations. In all scenarios, the population sizes of species *T* and *S* ($\theta_{T}$ and $\theta_{S}$) are estimated poorly when compared to those of species *R*$\left(\theta_{R}\right)$. This can be explained by the fact that more coalescence events occur in species *R* than in species *S* and *T*, leading to sequences containing more information about $\theta_{R}$. More precise results are expected with an increasing number of loci (Huang et al. 2020).

To figure out whether the superiority of BPP over the heuristic methods in detecting ghost introgression arises from its ability to effectively utilize branch-length information or from the use of a set of pre-constructed candidate models, we implemented the PhyloNet/ML method on true or inferred gene trees. This method searches the model space in the same manner as PhyloNet/MPL and is capable of incorporating gene-tree branch lengths. The results are presented in Supplementary Figure S6. When the true gene trees were used as input, PhyloNet/ML can accurately estimate introgression models and introgression probabilities γ, outperforming PhyloNet/MPL. This result highlights the crucial role of branch-length information in identifying network models. However, when it came to the inferred gene trees, the accuracy of network inference dropped dramatically, even to nil in the case of inflow. The poor performance of PhyloNet/ML on inferred gene trees confirms the previous finding that PhyloNet/ML is prone to gene tree estimation error (Yu et al. 2014; Cao et al. 2019). The developers behind PhyloNet also caution against utilizing gene-tree branch lengths in PhyloNet/ML unless one is certain of them (Cao et al. 2019). In contrast, the full-likelihood method BPP operates directly on sequence data and averages over the unknown gene-tree topologies and branch lengths to properly accommodate their uncertainties. Therefore, in practical terms, a full-likelihood approach is the only choice for detecting ghost introgression.

### Results of Ancient Introgression Scenarios

**Figure 5.**
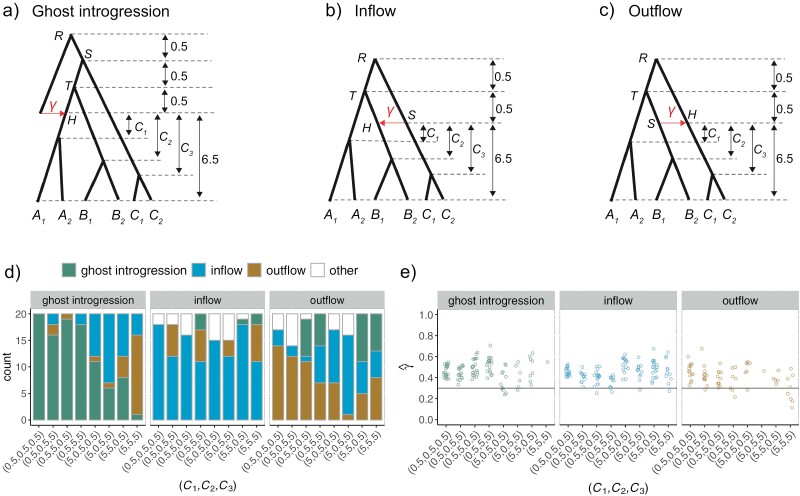
Results of PhyloNet/MPL in ancient introgression scenarios. a–c) The ancient scenarios of ghost introgression, inflow, and outflow, along with the corresponding parameter settings. $C_{i}\{i=1,\;2,\;3\}$ denote the time intervals of the introgression event and the divergence event of species *A*_*1*_ and *A*_*2*_ or species *B*_*1*_ and *B*_*2*_ or species *C*_*1*_ and *C*_*2*_, respectively, and were set to 0.5 or 5 coalescent units. The introgression probability γ was fixed at 0.3. d–e) Plots of results for network inference and γ estimation in simulations. The strips located on top of each plot represent the true scenarios. The *x*-axis is labeled with the parameter combination $\left(C_{1},\;C_{2},C_{3}\right)$. d) Estimation of network topologies. The bar chart illustrates the numbers of 3 inferred network topologies (denoted as topologies a–c) across 20 replicates. The remaining replicates that are not shown correspond to other inferred network topologies. e) Estimation of introgression probability γ. The solid line represents the true value, while the points depict the estimates within the true networks.

We also explored the performance of PhyloNet/MPL in detecting ancient ghost introgression. To do this, we simulated ancient introgression scenarios, wherein lineages undergo subsequent divergence events after an introgression event (Fig. 5a–c). We set the time intervals between the introgression event and the post-introgression divergence events (e.g. the divergence of *A*_*1*_ and *A*_*2*_) at 0.5 or 5 coalescent units to assess how this affects the performance of PhyloNet/MPL.

When the times between the introgression and divergence events are long, with $\left(C_{1},\;C_{2},C_{3})=(5,\;5,\;5\right)$, the 3 networks that signify ghost introgression, inflow, and outflow remain indistinguishable (Fig. 5d). When the introgression event is quickly followed by subsequent divergence events, with $\left(C_{1},\;C_{2},C_{3})=(0.5,\;0.5,\;0.5\right)$, the networks can be inferred with 100% accuracy in the cases of ghost introgression and with at least 70% accuracy in inflow and outflow cases. This is probably because a greater variety of gene-tree topologies ensue from the small time gap between the two kinds of events (see “Discussion” section for details). Unexpectedly, γ tends to be overestimated in all scenarios and most parameter combinations (Fig. 5e).

## Analyses of Empirical Data: *Jaltomata*

**Figure 6.**
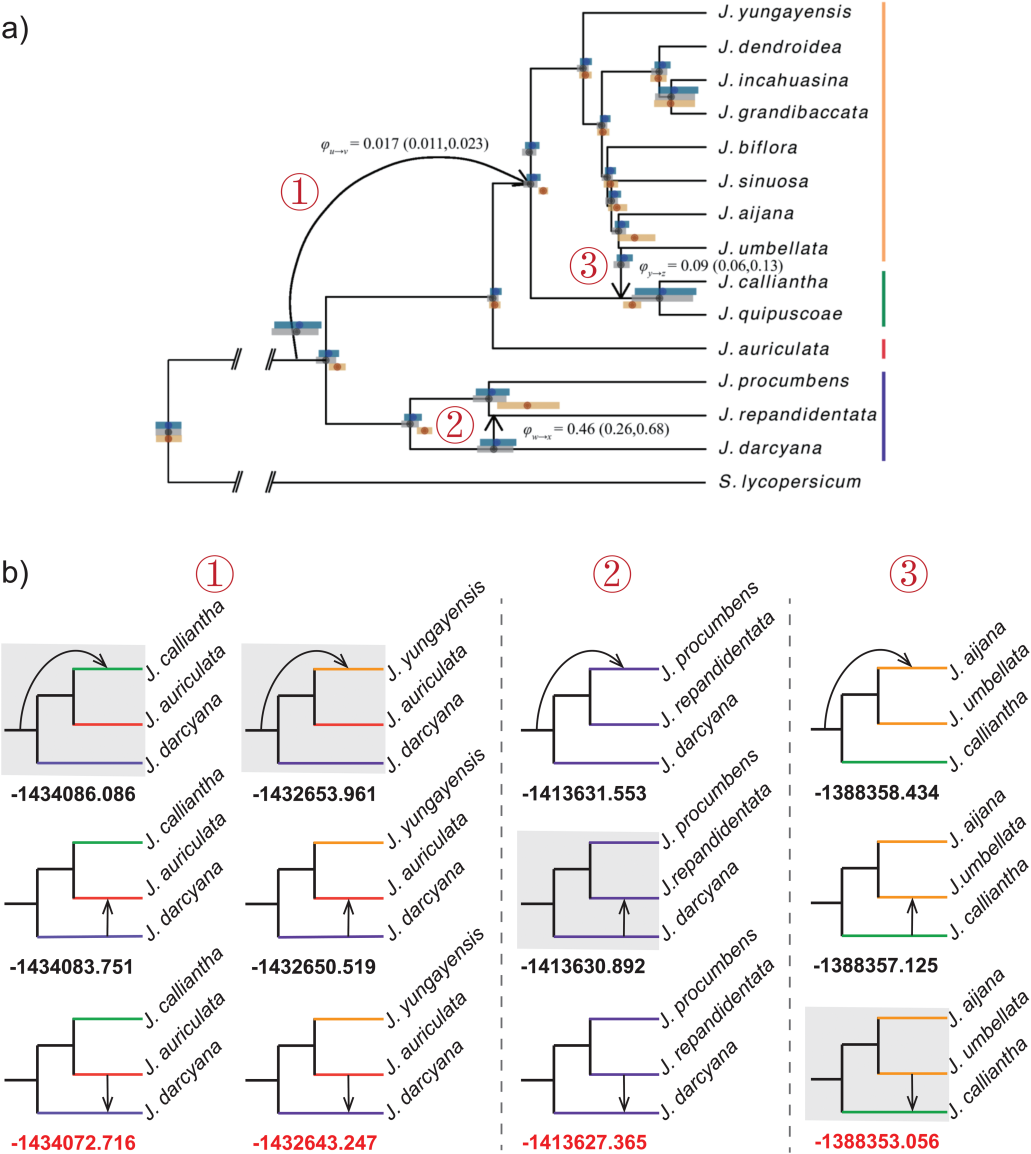
Inferred introgression events among *Jaltomata* species. a) The network topology inferred by SNaQ in Tiley et al. (2023; Figure 7) displays 3 introgression events. b) Comparison of ghost introgression, inflow, and outflow models for each introgression event using BPP. The models labeled by the gray shading align with the network topology in Figure 6a. Each model is marked with its corresponding log marginal likelihood value below, with the highest value indicating the best model.

To investigate whether the above findings apply to real-world cases, we reanalyzed the transcriptome data from fourteen *Jaltomata* species (Wu et al. 2018) and the outgroup *Solanum lycopersicum*. *Jaltomata* has experienced a recent radiation resulting in major subgroups with distinct mature fruit colors (Miller et al. 2011). Using the D-statistic, the original study by Wu et al. (2018) reported an ancient introgression event between the early diverging purple-fruited clade and the red-fruited lineage. A recent study by Tiley et al. (2023) first estimated a species tree using ASTRAL (Zhang, Rabiee, et al. 2018) and then identified 3 introgression events in *Jaltomata* using SNaQ (Solís-Lemus and Ané 2016): an ancient ghost introgression event from an unknown ancestral lineage of *Jaltomata* to the common ancestor of the green- and orange-fruited clades, an inflow event between the extant lineages within the purple-fruited clade, and an outflow event from orange-fruited *J. umbellata* to the green-fruited common ancestor (Fig. 6a).

Here, we revisit the 3 introgression events identified by the heuristic method SNaQ. As already alluded to above, SNaQ suffers from essentially the same unidentifiability issue as PhyloNet/MPL because they both maximize the pseudo-likelihood of a set of gene-tree topologies. We first investigated the ancient ghost introgression event from an unknown ancestral lineage of *Jaltomata* to the common ancestor of the green- and orange-fruited clades. We considered 30 possible combinations of 3 species from each of the purple-fruited clade, the red-fruited clade, and the subgroup comprising the orange- and green-fruited clades (Supplementary Tables 1 and 2). HyDe and PhyloNet/MPL were used to reanalyze each species trio. For HyDe, the concatenated sequence alignments for every species trio, along with the outgroup *Solanum lycopersicum*, were used as input, and the *P* value was calculated using the jackknife method, followed by B–H correction for multiple testing. For PhyloNet/MPL, we estimated maximum likelihood (ML) gene trees from 6431 one-to-one ortholog alignments using IQ-TREE v2 (Minh et al. 2020) with ModelFinder (Kalyaanamoorthy et al. 2017) to select the best-fitting substitution model and 1000 ultrafast bootstraps (-B 1000 -m MFP). These unrooted gene trees were then rooted with the outgroup *Solanum lycopersicum*. The subtrees for a focal species trio were extracted from the estimated gene trees as input for PhyloNet/MPL. Furthermore, due to the extensive computation required, BPP was used to analyze only 2 trios consisting of purple-fruited *J. darcyana*, red-fruited *J. auriculata*, and either green-fruited *J. calliantha* or orange-fruited *J. yungayensis*. To save computational time, we used only 1000 orthologous alignments to compare the 3 models of ghost introgression, inflow, and outflow under the backbone topology from Tiley et al. (2023) by calculating the marginal likelihood values with 16 quadrature points. For each of the 16 MCMC runs, we used 50,000 iterations for the burnin, after which we took 1,000,000 posterior samples, sampling every 2 iterations. We then conducted the A00 analysis to estimate parameters for the optimal introgression model. Each run took ~11 h using a single thread. The inflow event and the outflow event in Fig. 6a were investigated following the same procedure as just mentioned.

Our results on each of the three introgression events are presented in Fig. 6 and Supplementary Tables 1–3. First, let us look at the ghost introgression event, labeled as ➀ in Fig. 6a. According to the HyDe analysis, the inflow scenario involving red-fruited *J. auriculata* as the hybrid was supported by all considered trios, with the estimated introgression probability varying from 0.0236 to 0.0526 across different species trios (Supplementary Table 1). However, PhyloNet/MPL failed to distinguish between the three scenarios of ghost introgression, inflow, and outflow as their log pseudo-likelihood values were close to each other (Supplementary Table 2). The introgression probability estimated by PhyloNet/MPL ranged widely from 0.075231 to 0.486673 across different trios. As expected, BPP was able to distinguish between the 3 introgression scenarios. For both trios (*J. darcyana*, *J. auriculata*, *J. calliantha*) and (*J. darcyana*, *J. auriculata*, *J. yungayensis*), the log marginal likelihood values for the outflow model were greater than 7 compared to those for the ghost introgression and inflow models. In simpler terms, the Bayes factors favoring the outflow model over the other models exceed $e^{7}(\approx 1097)$, indicating that the outflow model exhibited a significantly superior fit to the observed data (Fig. 6b). Our results are consistent with the ancient introgression between red-fruited *J. auriculata* and the purple-fruited clade identified by the D-statistic in the original study by Wu et al. (2018) but contradict the conclusion of ghost introgression in Tiley et al. (2023). BPP estimated the introgression probability of the supported outflow event as 0.0584 and 0.05 for the 2 trios (Supplementary Table 3).

Next, we investigated the inflow event (labeled as ➁ in Fig. 6a) within the purple-fruited clade, considering the sole species trio of *J. procumbens*, *J. repandidentata*, and *J. darcyana*. The result of HyDe supported the inflow event with an introgression probability of 0.266 (Supplementary Table 1). Again, PhyloNet/MPL could not differentiate between the 3 scenarios of ghost introgression, inflow, and outflow, as their log pseudo-likelihood values were very close (Supplementary Table 2). In contrast, BPP favored the outflow scenario, as evidenced by the Bayes factors of the outflow model against the other two models exceeding 20 (Fig. 6b). This BPP result also contradicts the previous conclusion of an inflow event in Tiley et al. (2023). The introgression probability for the supported outflow event is estimated as 0.11 (Supplementary Table 3).

Finally, we examined the outflow event (labeled as ➂ in Fig. 6a) from orange-fruited *J. umbellata* to the green-fruited clade. We used HyDe and PhyloNet/MPL to analyze 14 different species trios, each involving *J. umbellata*, the green-fruited clade, and the orange-fruited clade. The HyDe results for all these trios supported an inflow event with *J. umbellata* as the hybrid (Supplementary Table 1). PhyloNet/MPL still could not distinguish between the three scenarios (Supplementary Table 2). The trio including *J. calliantha*, *J. umbellata*, and *J. aijana* was analyzed in BPP. The outflow model was favored, as indicated by the Bayes factors exceeding 20 against the other 2 models (Fig. 6b). This confirms the original conclusion of Tiley et al. (2023). The introgression probability was estimated to be 0.081, which is close to the original estimate of 0.09 in Tiley et al. (2023) (Supplementary Table 3).

## Discussion

Ghost introgression has garnered significant attention among evolutionary biologists in recent years. The studies conducted by Pang and Zhang (2023) and Tiley et al. (2023) have established the significant impact of ghost introgression on species tree inference and divergence time estimation. Tricou et al. (2022b) demonstrated that ignoring ghost introgression can lead to the misidentification of both donors and recipients of introgression events using the D-statistic. In this article, our aim is to determine the potential of currently popular phylogenetic tests for introgression in detecting ghost introgression or the pitfalls that may arise from failing to account for it.

### Unidentifiability of Ghost Introgression in Heuristic Methods

Based on theoretical analyses and simulation results, our study reveals that heuristic methods such as HyDe and PhyloNet/MPL cannot accurately detect ghost introgression, in the simplest cases of 3 focal species and a distant outgroup. This is because cross-species introgression introduces genealogical variation across independent loci, impacting the probabilistic distribution of gene-tree topologies and coalescent times (Yu et al. 2014; Flouri et al. 2020). Nonetheless, HyDe pools sites across the genome when counting site patterns, leading to the loss of important information contained in the variance of site-pattern counts across loci (Zhu and Yang 2021; Ji et al. 2023). PhyloNet/MPL only considers the distribution of gene-tree topologies without branch lengths. Consequently, analyses based on pooled site-pattern counts or gene-tree topologies can only indicate whether introgression has occurred, but cannot differentiate between different types of introgression such as ghost introgression, inflow, or outflow. Although both HyDe and PhyloNet/MPL face the same issue of network unidentifiability, they exhibit distinct patterns of behavior. Specifically, due to the assumption of hybrid speciation, HyDe’s interpretation is unequivocally oriented toward inflow cases when the statistically significant test confirms the presence of introgression. As such, HyDe works well only in inflow cases, but it can misidentify both the donor and recipient of introgression in both ghost introgression and outflow cases. By contrast, PhyloNet/MPL is liable to wrongly identify ghost introgression as ingroup introgressions and *vice versa*. Therefore, while HyDe and PhyloNet/MPL can provide indications of a specific introgression scenario, their conclusions should not be regarded as definitive. As with the D-statistic (Tricou et al. 2022b), the genuine interpretation of an introgression outcome when utilizing HyDe and PhyloNet/MPL should be a set of potential scenarios. That is, 3 scenarios including ghost introgression, inflow, and outflow, should all be given equal consideration.

The introgression probability γ is unidentifiable when using HyDe and PhyloNet/MPL to detect ghost introgression. In HyDe, the accuracy of γ estimation is determined by the ratio of 2 internal branch lengths, that is, $C_{T}$ and $C_{S}$ (or $l_{T}$ and $l_{S}$), which reflect the levels of ILS within the speciation and introgression histories, respectively (Fig. 2b). When $C_{T}=C_{S}$, γ can be estimated with high accuracy, even when HyDe makes an erroneous inference about the introgression scenario. In contrast to HyDe, the estimates of γ by PhyloNet/MPL are more dispersed across multiple replicates. The degree of dispersion also depends on both branch lengths $C_{T}$ and $C_{S}$ (Fig. 2a). Longer branch lengths (i.e., weaker ILS) can lead to more precise estimates of γ in PhyloNet/MPL.

In ancient introgression scenarios where lineage divergence events occur after the introgression event (Fig. 5a–c), identifying ghost introgression presents similar challenges when the time gap between introgression and subsequent divergence is significant. The challenges encountered by heuristic network inference methods can be attributed to the fact that sequences from recently diverged species have a high chance of coalescing within the long internal branch, making the introgression inference effectively the same as in the aforementioned 3-taxon cases. Conversely, if the introgression event is followed immediately by the divergence events, it can result in a greater variety of gene-tree topologies. This diversity imparts the essential information required for the accurate identification of ghost introgression or introgressions involving extant taxa (Zhu and Degnan 2017). The enhanced performance of PhyloNet/MPL in certain ancient introgression scenarios suggests that including additional species has the potential to improve the capability of heuristic methods in identifying introgression scenarios. A relevant observation by Pease and Hahn (2015) revolves around the effectiveness of a heuristic statistic known as *D*_FOIL_, which employs site pattern counts to identify introgression between non-sister species. In the context of a symmetric 4-taxon tree rooted with an outgroup, this statistic accurately identifies the introgression donor and recipient lineages. To clarify, our theoretical finding that heuristic methods based on gene-tree topologies or site-pattern counts cannot differentiate between the scenarios of ghost introgression, inflow, and outflow is constrained primarily to a rooted species triple (or an unrooted quartet); these introgression scenarios could become identifiable when more species are included in the analysis (Degnan 2018; Hibbins and Hahn 2022a; Thawornwattana et al. 2023).

### The Promise of Full-likelihood Methods in Detecting Ghost Introgression

Several studies have highlighted the statistical advantages of using full-likelihood methods, as opposed to heuristic methods, in species tree inference, parameter estimation, and gene flow detection (Xu and Yang 2016; Flouri et al. 2020; Jiao et al. 2021; Zhu and Yang 2021; Yang and Flouri 2022; Ji et al. 2023). Ji et al. (2023) have shown that the full-likelihood method BPP is more efficient than the heuristic method HyDe in detecting the presence of gene flow, regardless of whether the involved species are sisters or non-sisters. Additionally, as has been repeatedly pointed out, unidentifiable gene flow models when using heuristic methods may be identifiable when full-likelihood methods are applied to the same data (Jiao et al. 2021; Yang and Flouri 2022). Our research focuses on exploring the advantages of full-likelihood methods in distinguishing different introgression scenarios that are compatible with a significant D-statistic.

Most satisfyingly, full-likelihood methods are capable of identifying ghost introgression. These methods take into account the information of topologies and branch lengths in gene trees and can differentiate between ghost introgression and introgressions between non-sister species that are indistinguishable by heuristic methods using topologies alone. The importance of branch lengths in network identifiability has been underscored in Yu et al. (2012) and Zhu and Degnan (2017) as well. Additionally, including branch lengths in gene trees can render some model parameters identifiable, such as population sizes, species divergence times, and introgression probabilities, which can help researchers better understand the evolutionary histories of species.

A major drawback of full-likelihood methods is their considerable computational burden, restricting their application to a limited number of taxa. Therefore, we recommend using full-likelihood methods only when putative introgression events have been implied by heuristic methods, just as we have done in the above analysis of *Jaltomata*. Besides, while some MSC models in BPP have already relaxed the strict clock assumption (Flouri et al. 2022), the MSci model continues to assume it and thus is unsuitable for distantly related species (Flouri et al. 2020). In contrast, heuristic methods make use of frequencies of gene-tree topologies, thus they are robust against the violations of the molecular clock assumption. The final caveat is that detecting ghost introgression necessitates a known species tree, just like most phylogenomic methods for introgression inference (Hibbins and Hahn 2022a). However, it can be challenging, if not impossible, to accurately infer the species tree from sequence data due to extensive introgression affecting much of the genome (Mallet et al. 2016; Jiao et al. 2020). An alternative approach may involve utilizing genome-structure information, such as gene order or content, which is more resistant to the effects of gene flow. Incorporating these features into the inference of species tree topologies could provide a promising avenue for overcoming this challenge (Zhao et al. 2021; Ding et al. 2023).

### Implications for Real Data Analysis of Ghost Introgression

The simulation studies conducted by Tricou et al. (2022b, 2022a) suggest that ghost introgressions are probably more likely to occur than ingroup introgressions. In recent years, empirical evidence for ghost introgression has begun to appear in phylogenomic datasets such as those concerning *Jaltomata* species (Tiley et al. 2023) and on *Thuja* species (Li et al. 2022). However, it is notable that most such studies relied heavily on heuristic methods such as PhyloNet/MPL and SNaQ, as they are computationally tractable for large-scale genomic data involving more than a few taxa. In the present study, we have unequivocally demonstrated that these heuristic methods usually confuse ghost introgression and introgression between non-sister species. This raises doubts about the biological conclusions regarding introgression events drawn from studies that rely solely on these heuristic methods.

Our reanalysis of the empirical dataset from *Jaltomata* species showcases the limitations of heuristic methods in distinguishing between ghost, inflow, and outflow introgressions and highlights the promise of full-likelihood methods for accurately inferring ghost introgression. In our reanalysis of the 3 introgression events among *Jaltomata* species inferred by SNaQ (Tiley et al. 2023), HyDe consistently identified them as inflow introgression, while PhyloNet/MPL could not distinguish between the 3 introgression scenarios with very close pseudo-likelihood values. These results align perfectly with our theoretical analyses and simulation results. By comparison, the full-likelihood BPP analysis supported an outflow event from the red-fruited *J. auriculata* to the purple-fruited clade, rather than the previously suggested ghost introgression in Tiley et al. (2023), as well as favored another outflow event from purple-fruited *J. repandidentata* to *J. arcyana* instead of the previously proposed inflow event in the reverse direction (Tiley et al. 2023). In other words,2 out of the 3 introgression events inferred by SNaQ warrant further investigation.

We also reanalyzed the ghost introgression event among *Thuja* species as discovered by Li et al. (2022) (more details given in Supplementary Note 3 and Supplementary Table S4). Our results obtained using the selected species trio, once again indicated that HyDe and PhyloNet/MPL performed poorly, while BPP provided strong evidence in favor of the ghost introgression model. This conclusion about ghost introgression was also drawn from analyzing all *Thuja* species using PhyloNet/MPL in the original study. This firmly establishes the presence of ghost introgression among *Thuja* species, and we expect more properly confirmed cases of ghost introgression to come out in the future.

Here, we propose using the full-likelihood method BPP as a crucial step in validating gene flow events identified by heuristic methods. This involves comparing the focal gene flow event with 2 alternative introgression models through marginal likelihood calculation for relevant species trios, as demonstrated in our analyses of *Jaltomata* and *Thuja*. We have demonstrated that this strategy is both computationally feasible and effective in identifying ghost introgression. We recommend exercising caution when inferring introgression events using heuristic methods alone and suggest adopting a full-likelihood approach whenever possible to confidently detect ghost introgression in empirical studies.

## Conclusion

Our study highlights the issue of non-identifiability of ghost introgression in heuristic methods such as HyDe, PhyloNet/MPL (or SNaQ), which raises doubts about the reliability of previous conclusions on introgression that heavily or solely rely on these methods for inferring gene flow events. Therefore, we caution against interpreting heuristic method results without considering the possibility of ghost introgression and recommend adopting full-likelihood methods to make further judgments about the nature of the introgression event. This strategy is both computationally feasible and effective, making it the best practice for detecting ghost introgression in empirical studies. Undoubtedly, future efforts should be directed toward improving the statistical efficiency of heuristic methods and the computational efficiency of Bayesian MCMC algorithms employed in full-likelihood methods.

## Supplementary Material

Data available from the Dryad Digital Repository: https://doi.org/10.5061/dryad.zs7h44jfz
